# The protective effects and genetic pathways of thorn grape seeds oil against high glucose-induced apoptosis in pancreatic β-cells

**DOI:** 10.1186/1472-6882-14-10

**Published:** 2014-01-09

**Authors:** Xihu Lai, Xincong Kang, Luman Zeng, Jian Li, Yan Yang, Dongbo Liu

**Affiliations:** 1 Hunan Provincial Key Laboratory of Crop Germplasm Innovation and Utilization, Hunan Agricultural University, Changsha, Hunan 410128, P. R. China; 2 State Key Laboratory of Subhealth Intervention Technology, Changsha, Hunan 410128, P. R. China; 3 Hunan Agricultural University, Changsha, Hunan 410128, P. R. China

**Keywords:** Diabetes mellitus, Thorn grape seed oil, Anti-apoptosis, Insulin release

## Abstract

**Background:**

Excessive apoptosis of β-cell is closely related to diabetes mellitus. Chronic exposure to high glucose causes β-cell dysfunction and apoptosis in diabetes. Thorn grape (*Vitis davidii Foex.*) has been used to treat diabetes in Traditional Chinese medicine for many years. In our previous research, thorn grape seeds oil (TGSO) showed promising anti-diabetic effects in animal models. However, it is unknown whether TGSO played an anti-apoptotic role in the anti-diabetic effects and the mechanism regarding signal transduction pathway is unclear either.

**Methods:**

The rattus pancreatic β-cell line RIN-m5F was treated with/without TGSO which was extracted by supercritical carbon dioxide (CO_2_) fluid extraction and analyzed by Gas Chromatography/Mass Spectrometry (GC/MS). Cell apoptosis was detected by fluorescence activated cell sorting (FACS), insulin secretion was assayed by Enzyme-Linked Immunosorbent Assay (ELISA), and the apoptosis-related genes expressions were evaluated by quantitative Reverse Transcription Polymerase Chain Reaction (qRT-PCR).

**Results:**

TGSO, containing 87.02% unsaturated fatty acids (UFAs), significantly reduced pancreatic β-cell apoptosis and protected the insulin secretion impaired by high glucose. The expressions of pro-apoptotic genes such as *iNOS*, *Caspase-3*, *ATF-3*, *JNK*, *p38* and *Fas* were down-regulated while the anti-apoptotic genes *Akt* and *Bcl-2/Bax* were up-regulated.

**Conclusions:**

The results indicated that TGSO protected β-cells from high glucose-induced apoptosis and its protective activity may be linked to mitochondrial pathway, endoplasmic reticulum (ER) stress pathway and Fas signal pathway, which implied that TGSO might be an effective complementary or alternative medicine to reduce β-cell apoptosis and dysfunction.

## Background

Diabetes mellitus is characterized by hyperglycemia resulting from a relative or absolute insulin deficiency [1]. The prevalence of diabetes has risen dramatically during recent decades, and it is now a serious global health burden [2]. It is reported that more than 371 million people have diabetes (IDF Diabetes Atlas 5th Edition 2012 Update) with the biggest population in China, soaring to 92.3 million, 9.42% of adults (http://www.worlddiabetesday.org/). Insulin deficiency is the consequence of compromised insulin secretion per β-cell and/or reduction in total β-cell mass [3]. Prolonged exposure to high glucose concentrations exerts toxic effects on β-cell, resulting in β-cell dysfunction and ultimately apoptosis [4].

Evidences from both human disease and mouse models have shown that apoptosis is the main form of β-cell death in diabetes mellitus [3]. Beta-cell apoptosis is mainly mediated by three pathways, namely, death receptor signal pathway, mitochondrial pathway and endoplasmic reticulum (ER) stress pathway [5,6]. The death receptor signal pathway is activated upon ligation of the cell surface death receptors, including Fas receptor and tumor necrosis factor receptor-1 (TNFR-1), which in turn activates downstream effector of Caspase family [7]. The assembly of the death inducing signaling complex (DISC), a multi-protein complex consisting of Fas receptor, the adaptor protein Fas-associated death domain, and pro-Caspase-8 (pro-cysteinyl aspartate specific proteinase-8), is the initiating signal for the processing of pro-Caspase-8 to its active form [7]. TNF forms the DISC in the similar way [8]. Depending upon the activity of Caspase-8, the ensuing signal can activate either downstream Caspase cascade or the mitochondrial death pathway or Nuclear Factor-kappa B (NF-kB) for efficient execution [9-11].

Mitochondrial pathway could be activated by the unbalance of the proportion of pro-/anti-apoptotic proteins (such as Bax, Bad, Bcl-2, Bcl-xL), then release several apoptotic factors to cytoplasm, such as cytochrome-c, apoptotic protease-activating factor 1, apoptosis inducing factor, second mitochondria-derived activator of Caspase/direct inhibitor of apoptosis-binding protein with low PI, endonuclease G to induce apoptosis [12-18].

In the processing of ER stress pathway, some pro-apoptotic effectors are activated under ER stress, such as C/EBP homologous protein, c-Jun N-terminal kinase (JNK), activating transcription factor-3 (ATF-3) [19-21]. ER stress could also activate *p38* through Ca^2+^ signal way and induce apoptosis [20,22]. Besides, under ER stress, *Akt* gene could promote cell survival [23].

Finally, these three pathways converge to activate Caspase-3 to execute cell apoptosis [24-26]. In addition to the three pathway aforementioned, *iNOS* (inducible nitric oxide synthase) could be induced by environmental exposure leading to NO (Nitric Oxide) formation, which have a direct deleterious effects on β-cells [27,28].

Free fatty acids are important to pancreatic β-cell for glucose-stimulated insulin secretion [29,30]. It is reported that unsaturated fatty acids (UFAs) usually protect β-cell from glucose-induced apoptosis and impaired β-cell proliferation [31,32]. *In vivo* research showed that UFA could alleviate insulin resistance and promote insulin secretion, while *in vitro* studies have revealed that omega-6 polyunsaturated fatty acid (PUFA) can prevent chemically induced diabetes and attenuate the oxidant stress that occurs in diabetes mellitus [33,34].

Thorn grape (*Vitis davidii Foex.*), one of characteristic plants in Hunan province, P. R. China, has been used to treat diabetes in Traditional Chinese medicine for centuries. It is rich in UFA, resveratrol, oligomeric proantho cyanidins, squalene and superoxide dismutase, *et al*. In thorn grape seed oil (TGSO), UFA is mainly composed of linoleic acid (ω-6 PUFA) and oleic acid (monounsaturated fatty acid, MUFA) [35,36]. In our previous research, TGSO showed remarkable anti-diabetic effects in animal models [37]. However, its anti-diabetic mechanism has not been clarified.

This study aimed to evaluate the effects of TGSO on high glucose-induced rattus pancreatic β-cell apoptosis by detecting the cell apoptotic rate and insulin levels, and reveal its mechanisms regarding signal transduction pathway by measuring related genes expression.

## Methods

### TGSO extraction and fatty acids analysis

Thorn grapes (*Vitis davidii Foex.*), authorized by Cultivar Variety Examination and Approving Committee of Hunan Province (No: XPD010-2005), were picked from Mayang county, Huaihua city, Hunan province, P.R. China and deposited in nursery garden of Hunan Agricultural University. The seeds were collected and dried at 50°C for 24 h and then smashed into the powder of 40 meshes. TGSO was extracted by using supercritical CO_2_ fluid extraction machine (HA231-50-06, Nantong Hua’An Supercritical Extraction Co. Ltd.) at CO_2_ flow of 20 ~ 30 L/h, extraction pressure of 34.2 Mpa, extraction temperature of 39.3°C, separation pressure of 12 Mpa and separation temperature of 55°C for 95 minutes.

Fatty acid composition of TGSO were analyzed by GC/MS with a QP2010 gas chromatograph-mass spectrometer (Shimadzu, Japan), equipped with flame ionization detector (FID), Rtx-WAX capillary column (30.00 m × 0.25 mm × 0.25 μm). Samples (1 μL) were prepared following the protocols: TGSO (100 mg) were dissolved in 5 mL hexane, 5 mL sodium hydroxide-carbinol solution (0.5 mol/L), then vortex-mixed for 30 s and incubated in a water bath at 80°C for 30 min. After cooled to room temperature, 1 ml hexane and 1 ml deionized water were added, vortex mixed for 30 s and centrifuged for 10 min at 8000 revolutions per minute (RPM). The supernatant were transferred into a GC vial after filtered with 0.45 μm membrane (Shanghai Xingya purification material factory, Shanghai, China) and injected in split mode (1:10) with injector temperature of 250°C for GC/MS analysis. Helium was used as carrier gas at a flow rate of 1.02 mL/min. The oven temperature was 100°C for 3 min, and then increased from 100°C to 200°C at 5°C/min and at 200°C for 17 min. The detector operated in scan mode from m/z 40 to 500. Initial pretreatment of the raw chromatographic data, such as baseline correction, chromatogram alignment, noise reduction, and normalization, were performed by using GC/MS solution software. The peaks were then tentatively identified from their retention characteristics and mass fragmentation patterns by using NIST mass spectrum database.

### Cells culture and treatments

Rattus pancreatic β-cell line (Rin-m5F) was purchased from tumor cells bank of Chinese Academy of Medical Sciences. Rin-m5F cells were cultured at 37°C, 5% CO_2_ atmosphere in RPMI-1640 medium (Hyclone) containing 12% fetal calf serum (Hyclone). On attaining 75-80% confluency, cells (after passages 4) were seeded into 6-well plate at a density of 1 × 10^6^ per well and grown for 24 h. Then the cells were treated as different groups: control group (10 mM glucose), model group (25 mM glucose, 1000 μg/mL BSA (Bovine serum albumin)), 100 μg/mL TGSO group (25 mM glucose, 100 μg/mL TGSO, 1000 μg/mL BSA) and cultured for 48 h. The concentration of glucose, time for glucose exposure of RIN cells and TGSO concentration were obtained from our preliminary experiment. Rin-m5F cell was exposed to the glucose of 5, 15, 25 mmol/L for 12, 24, 36 and 48 hours. The treatment of exposure to 25 mmol/L glucose for 48 h was observed with amount of apoptotic cell and high NO content that it was appropriate for constructing high glucose-induced apoptosis model [38]. TGSO with the concentration of 50, 100, 200 μg/mL were set for cell culture. The results showed that 100 μg/mL TGSO played the best anti-apoptotic role by measuring apoptotic rate and insulin release. All cell cultures were set up in triplicates for following assay.

### Detection of cell apoptosis by fluorescence activated cell sorting (FACS)

Flow cytometric analysis was performed to detect the cell apoptosis of each group by using Annexin V-fluorescein isothiocyanate (FITC)/propidiumlodide (PI) kit (Qihaifutai Biotechnology, Shanghai, China) as recommended by manufacturer. After 48 h treatment, culture supernatants which contained floating dying and apoptotic cells were collected, and then merged with the adherent cells which were rinsed with phosphate buffered saline (PBS) and harvested by a brief trypsinization. The mixture was washed with PBS, centrifuged for 5 min at 2000 RPM, and the supernatants were discarded. Cells were re-suspended in 0.5 ml binding buffer, and then double-stained with FITC-conjugated Annexin-V and PI for 15 min at room temperature. Flow cytometric analyses were performed on a FACS Cytomics™ (FC500, Beckman Coulter, USA) within 1 h. The apoptotic cells measured as late or early apoptotic cells were shown respectively in the upper right or lower right quadrant of the FACS histogram.

### Insulin secretion assay

Insulin concentration in the supernatant which were collected after 48 h cultivation was determined by ELISA using microplate reader (MB-530, Shenzhen Huisong technology development Co., Ltd, Shenzhen, China). The measurement was conducted according to the instruction of Rat Insulin Elisa kit (R & D Systems, USA).

### Isolation of RNA, cDNA synthesis and qRT-PCR

Total RNA was isolated using the TRIzol reagent (CoWin Bioscience, Beijing, China). Its quality was assessed by electrophoresis on 1% agarose gels based on the integrity of 28S, 18S and 5S bands after ethidium bromide staining. The total RNA concentration was quantified by measuring absorbance at 260 nm and 280 nm. cDNA was synthesized from RNA by PrimeScriptTM RT reagent Kit with gDNA Eraser (TaKaRa, Japan) according to the instruction. qRT-PCR was performed on the CFX 96™ Real-Time System (C1000TM Thermal Cycler, BIO-RAD, USA). Primer sequences (Table 1) of *GAPDH*、*Caspase-3*、*iNOS*、*Bcl-2*、*Bax* and *Fas* were referred to our previous study [38] while the others (*ATF-3*、*JNK*、*p38*、*Akt*) were designed by Primer Premier 5.0 software (Applied Biosystems) with the cDNA sequences obtained from National Center of Biotechnology Information (NCBI) [GenBank: NM_012912.2, NM_053829.1, NM_031020.2, BF563829.1, respectively]. All of these primers were synthesized by Beijing Genome Institute (BGI-Shenzhen, China). For qRT-PCR, each reaction was run in triplicate and contained the following: 10.0 μL Premix Ex TaqTM II (Tli RNaseH Plus, 2 × Conc., TaKaRa, Japan), 0.8 μL Forward primer and Reverse primer (2.5 μM), 0.4 μL ROX Refrence Dye II (50 × Conc., TaKaRa, Japan) and 100 ng cDNA template in a final reaction volume of 20 μL.

**Table 1:** Primer sequences of target genes for qRT-PCR

**Target genes**		**Nucleotide sequences (5′-3′)**	**Product size**
*GAPDH*	Forward	AGCCCCCAACACTGAGCAT	111 bp
	Reverse	TGCAGCGAACTTTATTGATGGT	
*Caspase-3*	Forward	TTCTCCCTGGACGCCACTT	111 bp
	Reverse	CCTACCCCACTCCCAGTCATT	
*iNOS*	Forward	GTCTCTCCAAACCCCTCACTGT	112 bp
	Reverse	GGAGCAAAAAAGGGCAACAC	
*Bcl-2*	Forward	TGGGATGCCTTTGTGGAACT	125 bp
	Reverse	CAGGTATGCACCCAGAGTGATG	
*Bax*	Forward	GAGCTGCAGAGGATGATTGCT	128 bp
	Reverse	GCAAAGTAGAAGAGGGCAACCA	
*Fas*	Forward	GTGTGCAAGGCTCAAGGATGT	120 bp
	Reverse	TGGGATGCCTTTGTGGAACT	
*ATF-3*	Forward	TCTAGCCGCTCTCTGGACC	147 bp
	Reverse	TCCTCAAACACCAGTGACCC	
*JNK*	Forward	TCCAGTTCTCGTACCCGCTA	135 bp
	Reverse	AGCATGGCGTGACACAGTAA	
*p38*	Forward	TTTGCTCAGTACCACGACCC	107 bp
	Reverse	TCGTAGGTCAGGCTCTTCCA	
*Akt*	Forward	GCTGCGGCTCCTCATTCA	125 bp
	Reverse	CGCCGAGCTGGGAGTAAA	

After a pre-incubation step at 95°C for 30 s to activate DNA polymerase, PCR amplification was 40 cycles of denaturation at 95°C for 5 s and annealing at 60°C (except 64°C for *Akt*, *Fas*, *iNOS*) for 30 s. After amplification, the melting curves were generated in the range 65–95°C with increments of 0.5°C every 5 seconds. The emission data were quantified using the cycle threshold value. Data were normalized to *GAPDH* and presented as the mean fold change by comparing with the control group.

### Statistical analysis

The qRT-PCR data were analyzed by Livak 2^-△△Ct^ method [39]. Experimental result showed as mean ± standard deviation (SD). Statistical differences between the groups were analyzed by using the one-way analysis of variance (ANOVA). A value of p < 0.05 was considered significant and p < 0.01 represented highly significant.

## Results

### TGSO contained 87.02% UFA which was 5.7 folds higher than SFA (saturated fatty acid)

For identifying the functional composition in TGSO played the anti-diabetic role, its ingredients were analyzed by GC/MS as shown in Table 2. Twelve kinds of fatty acids were determined. Among them, the contents of UFA reached up to 87.02%, containing 20.16% MUFA and 66.86% PUFA. The rate of UFA and SFA was up to 670%.

**Table 2:** Composition and relative contents of fatty acids in TGSO

**No.**	**Retention time/min**	**Fatty acids**	**Relative content (%)**
1	17.355	Tetradecanoic acid	0.04
2	21.301	Hexadecanoic acid	7.73
3	21.757	11-Hexadecenoic acid	0.30
4	21.987	Ethyl 9-hexadecenoate	0.06
5	23.146	Heptadecanoic acid	0.08
6	25.233	Octadecanoic acid	4.92
7	25.695	9-Octadecenoic acid	18.91
8	25.845	11-Octadecenoic acid	0.69
9	26.924	9,12-Octadecadienoic acid	66.60
10	28.695	9,12,15-Octadecatrienoic acid	0.26
11	31.296	Eicosanoic acid	0.21
12	32.034	11-Eicosenoic acid	0.20

### TGSO reduced the percentages of apoptotic cells from 43.80% to 15.44%

**Figure 1:**
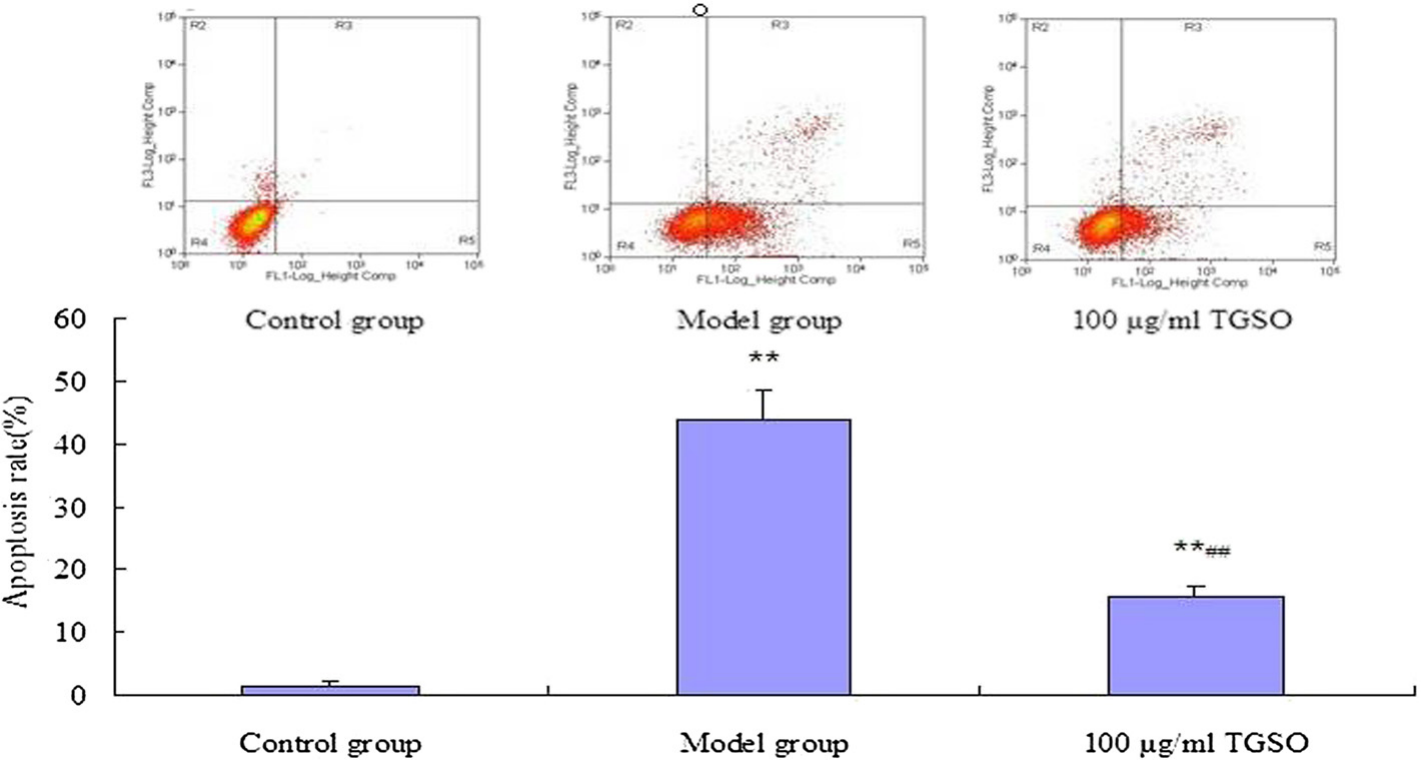
**Effects of TGSO on Rin-m5F Cells Apoptosis Induced by High Glucose.** Data represent mean ± S.D (n = 3 for each group). The values are statistically significant and presented as follows: **p< 0.01 vs. control group; ##p< 0.01 vs. model group.

To evaluate the effect of TGSO, the apoptotic rate of RINm5F cell exposure to high glucose with/without TGSO were investigated. After exposure to high glucose for 48 h, apoptotic rate of model group was significantly increased to 43.80%, which was 27.8-fold higher than that of the control. However, in the condition of co-culture with 100 μg/mL TGSO, the percentages of apoptotic cells were reduced to 15.44% (Figure 1).

### TGSO protected β-cells from insulin impairment induced by high glucose

**Figure 2:**
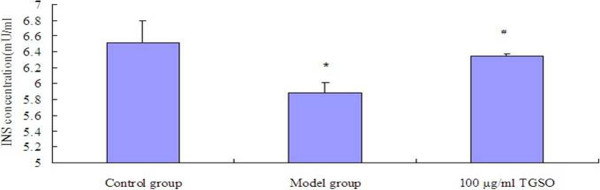
**Effects of TGSO on Insulin Secretion in Rin-m5F Cells Induced by High Glucose.** Data represent mean ± S.D (n = 3 for each group). The values are statistically significant and presented as follows: *p< 0.05 vs. control group; #p< 0.05 vs. model group.

As insulin release is one of the variables to assess high glucose-induced damage and protection afforded by TGSO, insulin concentrations of supernatant were decreased significantly after exposure to high glucose. TGSO recovered insulin secretion damage induced by high glucose to the comparable level of the control in Rin-m5F cells (Figure 2).

### TGSO down-regulated pro-apoptotic gene expression and up-regulated anti-apoptotic gene expression involved in signal transduction pathways

**Figure 3:**
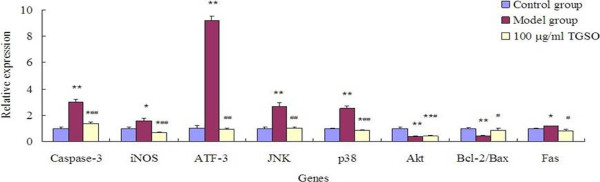
**Effects of TGSO on Apoptosis-Related Genes Expression in High Glucose- induced Rin-m5F Cells.** Data represent mean ± S.D (n = 3 for each group). The values are statistically significant and presented as follows: *p< 0.05 vs. control group; **p< 0.01 vs. control group; #p< 0.05 vs. model group; ##p< 0.01 vs. model group.

Related gene expression involved in three pathways was measured for anti-diabetic mechanism identification. After 48 h exposure to high glucose, mRNA expression levels of *Caspase-3*、*iNOS*、*ATF-3*、*JNK*、*p38*、*Fas* genes of model group increased to 298%, 159%, 917%, 264%, 254% and 119%, respectively, while *Akt*, *Bcl-2/Bax* genes reduced by 63% and 60% (Figure 3).

In TGSO group, the expressions of pro-apoptotic genes such as *Caspase-3*, *iNOS*, *ATF-3*, *JNK*, *p38* and *Fas* gene were down-regulated to 46%, 41%, 11%, 40%, 34% and 68% of the model group. Among them, expressions of *iNOS* and *p38* were even lower than the controls. The anti-apoptotic gene *Akt* and *Bcl-2/Bax* gene expressions were up-regulated to 110% and 213% compared with model group (Figure 3).

## Discussion

TGSO showed remarkable anti-diabetic effects in animal models, however, functional components in TGSO and its anti-diabetic mechanism have not been identified. In this investigation, the compositions of TGSO were analyzed by GC-MS and its mechanism was illustrated by measuring apoptotic rate, insulin release and related gene expression.

Glucose with supraphysiological level has deleterious effect on pancreatic β-cell function and consequently results in dysfunction and apoptosis [4]. As in our previous study, 25 mM glucose was used in this investigation to construct apoptotic cell models with high apoptotic rate and low insulin secretion [38]. Mechanisms of high glucose induced β-cell apoptosis include increasing iNOS expression together with regulation of apoptosis-related gene expressions involved in three pathways. In consistent with our previous observation, high glucose induced apoptosis via Fas signal pathway and imbalance expression of *Bcl-2* versus *Bax*. The anti-apoptotic gene *Bcl-2* expression did not response to high glucose stimulation, whereas pro-apoptotic *Bax* gene was increased [38], leading to the alteration of the mitochondrial homeostasis, and subsequent activation of apoptosis. In addition, up-regulated expressions of *ATF-3*, *JNK*, *p38* and down-regulated expressions of *Akt* revealed the high glucose induced apoptosis is closely related with ER stress pathway too. This is agreed with the review of Bensellam *et al.* that ER stress pathway plays an important role in β-cell glucotoxicity [4]. Among these tested genes in ER stress pathway, the modification of transcription factor *ATF-3* expression in model group is the highest (9.17 folds). This might due to its role as a downstream target of both NF-kB and JNK/SAPK signaling pathways and the induction of NO which is synthesized by iNOS [21].

After co-culture with TGSO, the cells were protected against high glucose-induced apoptosis and insulin secretion was reserved [31]. This may due to the UFA it contained with the ratio of UFA/SFA up to 670%. Though SFA was reported to induce apoptosis by mitochondrial dysfunction and production of reactive oxygen species [40,41], UFA could protect RINm5F cells against SFA-induced cell death, not only induction of apoptosis prevented [32], but also β-cell proliferation promoted [42]. It is reported that the anti-apoptotic UFA displays considerable structural specificity and it appears to regulate a signaling pathway that controls the activity of effector caspase enzymes in the cells [43]. UFA protection from apoptosis mediated through a PI3-kinase-dependent signaling pathway, since PI3-K inhibitor abolished the UFA protection [32]. These were supported by our observation that treatment of β-cell with TGSO causes attenuated expression of caspase-3 and increased expression of Akt, the downstream targets of PI3K.

The regulation of apoptosis-related gene expressions suggested that the protect activity of TGSO was via all of the three pathways, namely ER stress pathway, mitochondrial pathway and Fas signal pathway. In ER stress pathway, ATF3, as a member of the ATF/cAMP response element binding protein (CREB) family of transcription factors, its induction was reported to play an important role in β-cell destruction [44,45]. Li *et al.*[20] found that ATF-3 down-regulate the expression of insulin receptor substrate 2 (IRS2) while Allen-Jennings *et al.*[46] demonstrated its inhibition of β-cell proliferation. Hartman *et al.* speculated that ATF3 may in turn affect the ability of NO to modulate the cell death machinery directly or indirectly [21,47]. Burak Kutlu *et al.* observed that iNOS inhibitor reduced cytokine-induced ATF-3 expression in INS-1β cell [48]. In this investigation, the extreme high expression of *ATF-3* gene induced by high glucose was reduced to the comparable level of control which may be a result of declined NO formation. Besides, the *p38* expression might illustrate that the anti-apoptotic action played in ER stress pathway was mainly through the *p38* branch rather than *JNK*. In view of the cytochrome c release block and Bcl-2 expression decrease, mitochondrial pathway was reported to contribute to the protection of MUFA against saturated fatty acid-induced apoptosis in human β-cells [31]. In this research, the expression of Bcl-2/Bax was declined which implied that the protection of TGSO, containing 87.02% UFA, against high glucose was via mitochondrial pathway. In addition to these two pathways, the down-regulation of *Fas* gene revealed that Fas signal pathway was involved in this anti-apoptotic activity as well.

## Conclusions

TGSO protected the Rin-m5F β-cell from high glucose-induced β-cell apoptosis. Its anti-apoptotic action was mediated via ER stress pathway, mitochondrial pathway and Fas signal pathway. ATF-3 might play an important role in the anti-apoptotic activity of TGSO.

## Abbreviations

TGSO: Thorn grape seeds oil; CO2: Supercritical carbon dioxide; GC/MS: Gas Chromatography/Mass Spectrometry; ELISA: Enzyme-Linked Immunosorbent Assay; qRT-PCR: Quantitative reverse transcription polymerase chain reaction; SFA: Saturated fatty acids; UFA: Unsaturated fatty acids; PUFA: Polyunsaturated fatty acid; MUFA: Monounsaturated fatty acid; ER: Endoplasmic reticulum; TNFR-1: Tumor necrosis factor receptor-1; DISC: Death inducing signaling complex; NF-kB: Nuclear Factor-kappa B; JNK: c-Jun N-terminal kinase; ATF-3: Activating transcription factor-3; iNOS: Inducible nitric oxide synthase; NO: Nitric oxide; RPM: Revolutions per minute; BSA: Bovine serum albumin; FACS: Fluorescence activated cell sorting; PBS: Phosphate buffered saline.

## Competing interests

The authors declare that they have no competing interests.

## Authors’ contributions

DBL conceived this study, participated in its design and coordination, and revised the manuscript. XHL undertook the TGSO extraction, cell culture and statistical analysis, and drafted the manuscript. XCK undertook the qRT-PCR and drafted the manuscript. LMZ, JL participated in the cell culture and TGSO extraction, respectively, while YY designed the primers. All authors read and approved the final manuscript.

## Pre-publication history

The pre-publication history for this paper can be accessed here:

http://www.biomedcentral.com/1472-6882/14/10/prepub
